# A hybrid AI framework for identification of power quality disturbances in electrical network

**DOI:** 10.1038/s41598-026-35376-x

**Published:** 2026-04-02

**Authors:** Rajesh Debnath, Amitabha Majumder, Arvind Kumar Jain, Bishwajit Dey

**Affiliations:** 1 Department of Electrical Engineering, National Institute of Technology Agartala, Agartala, India; 2 Department of Electrical Engineering, Manipal University Jaipur, Jaipur, Rajasthan India

**Keywords:** Smart grid, Power quality disturbance, S-transform, Chi-square test, Long short term memory, Energy science and technology, Engineering, Mathematics and computing

## Abstract

Sustaining power quality is an utmost priority for energy distributors in a modern power system integrated with distributed generation and nonlinear loads. The power quality disturbances (PQDs) comprising of multiple PQD events creates complexities in accurate detection and classification. Therefore, this study presents an effective, resilient and automated hybrid classifier for detection and classification of complex power quality disturbances for single occurrence as well as combinations of double, triple, and quadruple occurrences. To develop an effective and cohesive classifier, it is vital to identify the applicable features from the disturbance signal that can enhance data efficiency while capturing the signal’s fundamental qualities. Consequently, a set of features is retrieved utilizing the Stockwell Transform (ST), and the significant features are identified by the Chi-Square Test (CST). These features are utilized to train the Long Short-Term Memory (LSTM) network. The method has been validated on the signals generated using Power System Computer Aided Design (PSCAD) and OPAL-Real Time (OPAL-RT) for the IEEE 9-bus and 33-bus systems. The novelty of proposed hybrid method lies in the real time validation under noisy conditions. Additionally, to signify the superiority of the suggested method, the performance of the classifier has been compared with a multi-layer feedforward neural network and existing approaches documented in the manuscript. The proposed hybrid method given 99.11% accuracy which is higher in comparison of existing methods. The results confirm the superiority of the suggested hybrid technique for the real-time detection and classification of PQD signals.

## Introduction

The voltage, current and frequency must follow certain predefined specifications for safe, reliable, economic and efficient operations. However, due to interaction between system and loads, voltage and current deviate from pure sinusoidal form which can be visualized as power quality problem. According to the utility point of view, power quality is reliability; on the other hand, from load side, it is the electrical power supplied for satisfactory performance of all equipment including sensitive ones. Currently, variety of non-linear loads including power electronic devices are extensively employed in industrial, commercial, and residential sectors. The advancement of technology has led to an increased utilization of sensitive equipment such as SMPs, PLCs, mobiles, intelligent control, and relaying devices. These devices exacerbating pollution in electrical systems due to their highly nonlinear characteristics^1^. Power Quality Disturbance (PQD) is any sort of variation which occurs in voltage, current or frequency, resulting in failure or mis operation of end utility i.e. electrical gadgets due to violation of normal operating conditions^2,3^. This poor PQ leads to several issues such as mal-operation of protection devices, over-heating of equipments’, and may lead to their failure also.

Rigorous study has been carried out to identify different types of PQDs. These disturbances get aggravated when harmonics come into the picture^4^. Hence, the monitoring and categorization of PQDs in the voltage or current waveforms is the first and the foremost step in alleviating effect of these problems. Consequently, an intelligent and self-regulated technique is highly appreciated for PQD classification in the present situation. In order to keep electrical power within the established normal regulations, it is crucial to detect and classify power quality disturbances.

A comprehensive review of AI and signal processing approaches for detection of PQDs has been presented in^5^. Signal processing approach, like Fourier Transform^6^, Short-time Fourier Transform^7^, Wavelet Transform^8^, S-Transform^9^, Wigner Ville Distribution^11^, Empirical Mode Decomposition^12^, Variational Mode Decomposition^13,14^ and Independent Component Analysis^15^ are in use for feature extraction. FT is the basic DSP technique, simple to implement but not suited for non-stationary signals because of inadequate time and frequency localization ability. STFT can analyze stationary and non-stationary signals both by utilizing a sliding window function and obtains the time-frequency information; though this technique is restrained due to the size of the sliding window. WT might be an alternative to emphasize the time and frequency resolution during the analysis; but it is noise-sensitive. The ST is the generalization of the STFT and extension of WT; therefore overcomes a few of the STFT and WT drawbacks and its working doesn’t get violated due to system-noise, making it superior to the FT, STFT, and WT methods. However, ST suffers from a high computational complexity. Nowadays, the WVD and EMD techniques have received considerable attention in doing analysis of non-stationary signals because of high time-frequency resolution and efficient performance in noisy conditions. Due to mode mixing and boundary effects, VMD is much effective and vigorous method for signal processing. The signal processing methods discussed above are frequently employed to extract features from various types of PQDs.

To segregate and automate the classification of PQDs in different classes, a variety of Artificial Intelligence (AI) algorithms are used. These algorithms include Artificial Neural Network^16^, Probabilistic Neural Network^17^, Support Vector Machine^18^, Extreme Learning Machine^19^, Decision Tree^20^ which comes predominantly under the machine learning algorithms. Deep Neural Network based classification techniques that rely less on distinct analytical models that excels autonomously discovering features within datasets can effectively tackle these problems. There are two primary trajectories for DL based methodologies. The literature suggested in the^44^ gives a useful insight about the application of DL based algorithms used for the classification of PQDs. The objective is to transform the one-dimensional sampling sequences of PQDs into two-dimensional images using visualization techniques, and to integrate these images with DL based classification approach to extract feature vector for the classification task. In^31^, the 1D PQD signal is transformed into a 2-D grayscale image, and the features are augmented using image enhancement algorithms. To emphasize the local and spatial characteristics, the Wigner–Ville distribution^32^, Gramian Angular Field (GAF)^33^ are employed to visualize the PQD events. DL based image categorization techniques can readily recognize visualized PQDs. The primary focus of these strategies is to optimize the characteristics of PQDs in the two-dimensional perspective.

Another approach involves using 1-D PQ events are sampled and fed as model input, exploiting both spatial feature extraction space of convolutional neural networks and temporal retention abilities of recurrent neural networks to classify power quality disturbances (PQDs). Wang et al.^34^ introduced a deep convolutional neural network, comprising multiple units, to automatically process one-dimensional sequences and extract complex high-dimensional characteristics for precise categorization. Gong and Ruan^35^ further enhanced this accuracy with an advanced DCNN that utilizes one dimensional modified inception-residual network (1-D MIR) algorithm for feature extraction.

Simulation results demonstrate that this model offers both increased convergence rates and superior generalization capabilities. Zu and Wei^36^ proposed a simple gated recurrent network that takes into account PQDs’ temporal relationships for processing purposes. In comparison to RNNs, long short-term memory^21–23^, Gated Recurrent Unit systems contain less parameters, thereby reducing computational demands while maintaining accuracy. It is observed that some of the literatures focuses on the conversion of 1-D PQD signal into 2-D images using different approaches like GAF, recurrence plot (RP), and mathematical morphological methods which are integrated through a correlation-driven feature decomposition fusion (CDDFuse) approach^45^. This conversion from 1-D signal to 2-D signal are done to map the spectral features from the PQD signal. This spectral feature contains the time-frequency information and these features are used as the input to the DL algorithms for automatic classification. Contrary to these approaches, classification algorithms that utilize 2-D images as input can intricate network architecture and dataset, leading to feature loss. With more complex PQDs being immerged due to grid modernization and interconnected nonlinear appliances, articulating unique characteristics within images has become more challenging than ever. By contrast, explicitly inputting 1-D signals into DL networks can more effectively classify the PQD events. Graph neural networks (GNNs) have emerged as a powerful technique for power quality assessment, due to their ability to capture spatial and topological relationships inside electrical distribution systems^46^. Nevertheless, their advantages, GNNs sometimes encounter heightened computational complexity, scalability challenges in large networks, and vulnerability to the quality of graph construction.

It is observed that some of the reported methods are unable to detect complex combinations such as sag with harmonics or swell with harmonics or higher combinations. Most of the reported methods require higher training and testing time for detection and classification of PQD events. Further, most of the researchers segregated up to two simultaneously occurring PQ disturbances. A few researchers discussed about the triple simultaneously occurring PQ disturbances. However, we do not come across any work which has reported classification of simultaneously occurring quadruple PQ disturbances.

To address the aforementioned deficiencies, it is essential to devise and implement a precise, efficient, and automated PQ detection methodology. This work’s innovation is in comparing several classification methods with the proposed ST-LSTM based hybrid technique, which is trained, validated, and evaluated using simulated and generated power quality disturbance events in a real-time platform. The key contributions made by the research are summarized below:

- An efficient hybrid classifier, integrating S-transform and LSTM network, is established to identify and categorise simultaneously occurring PQD events up to quadruple instances.
- Features of the PQ signal have been extracted using S-transform and Chi-square test has been used to select significant features which improves the performance and accuracy of the hybrid classifier.
- To validate the efficacy of the suggested classifier, its outcomes have been compared with the results obtained by MLFFNN, ST-PNN, ST-DHM, KF-Fuzzy, Adalin-FFNN, SSD-DT, TQWT-MSVM-RBF, CNN, DWT-RF, URPM-CWT, SSPQDD and the findings documented in Table 8.
- The efficacy of the suggested hybrid classifier has been validated in a real-time scenario utilising OPAL-RT.

This document is structured into four distinct sections. Section II presents the framework for analyzing power quality concerning various disturbance signals. The results of the suggested technique are detailed in Section III, and finally the conclusion is summarized in Section IV.

## Suggested methodology

Signals exhibiting various power quality disturbances can be analytically characterized as non-stationary signals due to their change in spectral behaviour with respect to time. To determine the types of PQDs present in a specific non-stationary signal, optimal features are initially extracted from a range of options and subsequently classified with precision. In this manuscript, decomposition of the required PQD signal have done using S-Transform, after that significant features are selected for classification by Chi-Square test, and segregation in different classes is carried out using MLFFANN and LSTM. The following sections provide an explanation of the same.

### S-transform (Stock well Transform)

The traditional spectrum analysis techniques are built on the concept of Fourier analysis, which relies on the assumption of signals being stationary; i.e., their statistical properties are time invariant. In practical systems, signals available in the power system are of finite-duration and non‐stationary. In the contrast, the S-transform serves as a time-frequency analysis technique, integrating the properties of the Short Time Fourier Transform and Wavelet Transform, making it apt for analyzing time-varying signals. It can offer frequency resolution while preserving a direct correlation with the Fourier spectrum. The S-Transform of a signal x(t) is expressed as^1^,

1 $$\:ST\left(T,F\right)=\underset{-\infty\:}{\overset{+\infty\:}{\int\:}}x\left(t\right).W\left(T-t,F\right).{e}^{-\left(j2\pi\:Ft\right)}dt$$

where, $\:W(T,F)$ is the Window Function. This type of Window functions, often referred to as weighting functions, tapering functions, or apodization functions, utilized in digital signal processing techniques, are mathematical functions that assume a value of zero outside a specified interval. The scalable Gaussian window function is defined as:

2 $$\:W\left(T,\delta\:\right)=\frac{1}{\sqrt{2}\delta\:\left(F\right)\pi\:}{.e}^{-\frac{{T}^{2}}{2{\left(\delta\:\left(F\right)\right)}^{2}}}\:$$

$\:\delta\:\left(F\right)\:$is called the Window Width which can be expressed as the standard deviation of that Window Function as:

3 $$\:\delta\:\left(F\right)=\frac{1}{\left|F\right|}\:$$

In this model, we maintain the window function identical to that of the Gaussian Window, as it fulfills the minimum requirement of the uncertainty principle. We introduced an extra parameter µ, representing the set of parameters that define the shape and characteristics of the Gaussian Window Functions. The window width fluctuates with frequency as detailed below.

4 $$\:\delta\:\left(F\right)=\frac{\mu\:}{\left|F\right|}$$

Therefore, the Window Function can be rewritten as

5 $$\:W\left(T,\delta\:,\mu\:\right)=\frac{\left|F\right|}{\sqrt{2}\mu\:\pi\:}{.e}^{-\frac{{T}^{2}{F}^{2}}{2{\mu\:}^{2}}}\:$$

Hence, the generalized S-transform of the signal $\:x\left(t\right)$ is expressed as

6 $$\:ST\left(T,F,\mu\:\right)=\underset{-\infty\:}{\overset{+\infty\:}{\int\:}}x\left(t\right).\frac{\left|F\right|}{\sqrt{2}\mu\:\pi\:}.{e}^{-\frac{{\left(T-t\right)}^{2}{F}^{2}}{2{\mu\:}^{2}}}.{e}^{-\left(j2\pi\:Ft\right)}dt$$

This Window Function must satisfy the normalized condition

7 $$\:\underset{-\infty\:}{\overset{+\infty\:}{\int\:}}W\left(T,F,\mu\:\right)dt=1$$

The discrete variant of the ordinary S-Transform, specifically. The computation of the Discretized S-Transform (DST) leverages the advantage of the Fast Fourier Transform in conjunction with the Convolution Theorem. By discretizing the continuous time domain signal x(t) with a sampling time interval of $\:\:{T}_{s}$, the resulting output will be the discrete time signal $\:x\left(n.\:{T}_{s}\right)$, where $\:n=\mathrm{0,1},2,\dots\:\dots\:,N.$ Assuming $\:T\to\:k.\:{T}_{s}$ and $\:F\to\:\frac{p}{N.{T}_{s}}$, the DST of the discrete signal can be expressed as

8 $$\:S\left[k.{T}_{s},\frac{p}{N.{T}_{s}}\right]=\sum\:_{q=0}^{N-1}X\left[\frac{p+q}{N.{T}_{s}}\right].G\left(p,q\right).{e}^{-\left(j\frac{2\pi\:qk}{N}\right)}$$

Where $\:X\left[\frac{p}{N.{T}_{s}}\right]$ is the Discrete Fourier Transform of $\:x\left(n.\:{T}_{s}\right)$, which can be formulated as

9 $$\:X\left[\frac{p}{N.{T}_{s}}\right]=\frac{1}{N}.\sum\:_{n=0}^{N-1}x\left(n.\:{T}_{s}\right).{e}^{-\left(j\frac{2\pi\:pn}{N}\right)}$$

Where $\:p,k=\mathrm{0,1},2,\dots\:\dots\:,\left(N-1\right)$ and $\:G\left(p,q\right)={e}^{-\frac{2{\pi\:}^{2}{q}^{2}}{{p}^{2}}}$is the Gaussian Function, which is same as that of the average of the time signal. Therefore, the DST can be rewritten as

10 $$\:S\left[k.{T}_{s},\frac{p}{N.{T}_{s}}\right]=\frac{1}{N}.\sum\:_{q=0}^{N-1}\sum\:_{n=0}^{N-1}x\left(n.\:{T}_{s}\right).{e}^{-\left(j\frac{2\pi\:\left(p+q\right)n}{N}\right)}.{e}^{-\frac{2{\pi\:}^{2}{q}^{2}}{{p}^{2}}}.{e}^{-\left(j\frac{2\pi\:qk}{N}\right)}$$

For $\:p=0$, the Discrete S-Transform $\:S\left[k.{T}_{s},0\right]$ is defined as

11 $$\:S\left[k.{T}_{s},0\right]=\frac{1}{N}.\sum\:_{q=0}^{N-1}X\left[\frac{q}{N.{T}_{s}}\right]$$

For better understanding of the above-mentioned method, the algorithm can be explained as:

(i) Apply Discrete Fourier Transform on the time signal $\:x\left(n.\:{T}_{s}\right)$ (having $\:N$ sampling points and a sampling time interval of $\:{T}_{s}$) to get $\:X\left[\frac{p}{N.{T}_{s}}\right]$ using Fast Fourier Transform technique and this is done for onetime.
(ii) Calculate the localizing Gaussian Function $\:G\left(p,q\right)$ for the required frequency of $\:\frac{p}{N.{T}_{s}}$.
(iii) Shift the spectrum $\:X\left[\frac{p}{N.{T}_{s}}\right]$ to $\:X\left[\frac{p+q}{N.{T}_{s}}\right]$ for the frequency of $\:\frac{p}{N.{T}_{s}}$.
(iv) Multiply $$\:X\left[\frac{p+q}{N.{T}_{s}}\right]\:\mathrm{b}\mathrm{y}\:G\left(p,q\right).$$.
(v) Inverse Fourier transform of the above to get row of $\:S\left[k.{T}_{s},\frac{p}{N.{T}_{s}}\right]$ corresponding to the frequency, $\:\frac{p}{N.{T}_{s}}$.
(vi) Reiterate steps iii, iv, and v until every row of $\:S\left[k.{T}_{s},\frac{p}{N.{T}_{s}}\right]$ matching to the defined discrete frequencies, $\:\frac{p}{N.{T}_{s}}$, that have been defined.

### Chi-square test

A Chi-Square test is utilized to assess the independence of two events. For the provided data set comprising two variables, we can determine the observed count, B, and the expected count, A. Chi-Square quantifies the deviation between expected count (A) and observed count (B)^10^. The formula applicable in the Chi-Square test can be defined as

12 $$\:{\chi\:}_{k}^{2}=\sum\:_{i=1}^{N}\frac{{\left({B}_{i}-{A}_{i}\right)}^{2}}{{A}_{i}}$$

In this context, N represents the sample size, k is defined as N-1, indicating the degrees of freedom, which refers to the greatest number of logically independent values that can fluctuate. $\:{B}_{i}$ and $\:{A}_{i}$ denote the observed and expected values of the ith variable, respectively, in this analysis. When two features are independent, the observed count approximates the expected count, resulting in a significantly smaller Chi-Square score. A high Chi-Square score signifies that the independence hypothesis is invalid. In other way the higher Chi-Square score indicates greater dependencies of the features on the response, making it appropriate for model training^6^.

The necessary procedures to do the Chi-Square test are outlined as follows.

- Define the Hypothesis (H_0_ and H_1_).
- Creating a Contingency table.
- Locating the expected values.
- Calculation of Chi-Square value.

Acceptance or Denial of the zero Hypothesis based upon the above Chi-Square value.

### Long short-term memory

**Fig. 1:**
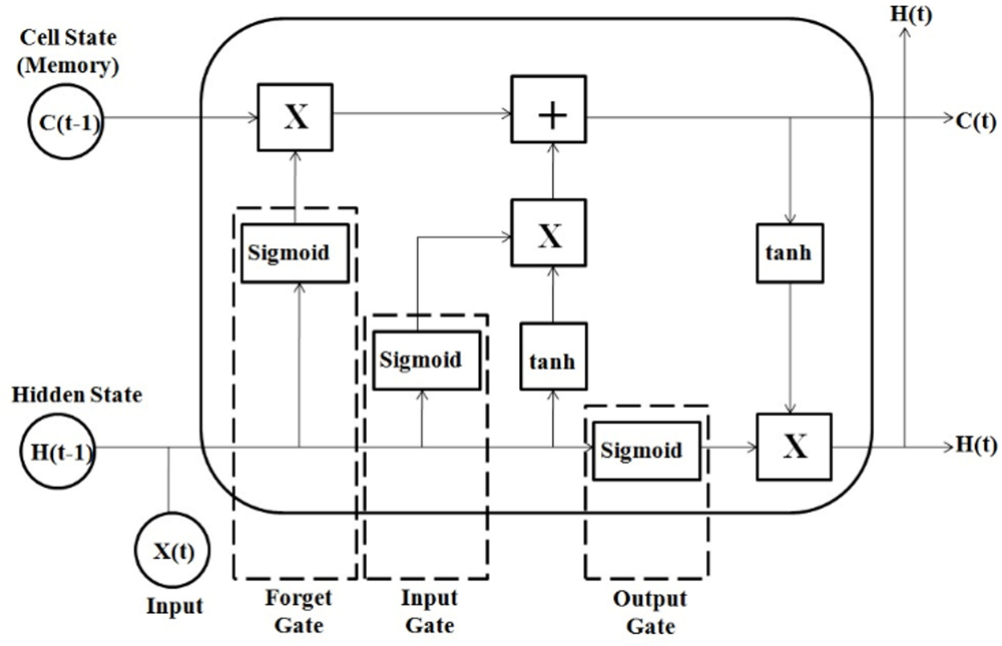
Architecture of long short-term memory network.

Long Short-Term Memory represents a distinct category of RNNs specifically discovered to solve the challenges associated with long-term dependencies that are prevalent in standard RNNs^22^. The primary benefit of LSTM lies in its ability to retain information over extended periods, enhancing its performance compared to other AI methods. Figure 1 illustrates the structure of LSTM.The architecture of Long Short-Term Memory consists of an input, output and several hidden layers. The memory node, adept at preserving its overall state time, incorporates a nonlinear operating method that regulates the flow of feature data inside and outside of the node, and is integrated within the hidden layer. Memory node which holds input gate, forgetting gate and output gate replaces the hidden layer neurons of a RNN network and helps to erase erroneous data or store critical data at each time step. Further, input gate behaves like a filter and blocks the irrelevant input. The forget gate supports to focus on the new information received and forget previously stored information in its memory. The output gate has sigmoidal activation function and provides output in 0,1 binary form to limit the gate output. It is answerable to reveal or not reveal the contents of the memory node at the LSTM.

**Fig. 2:**
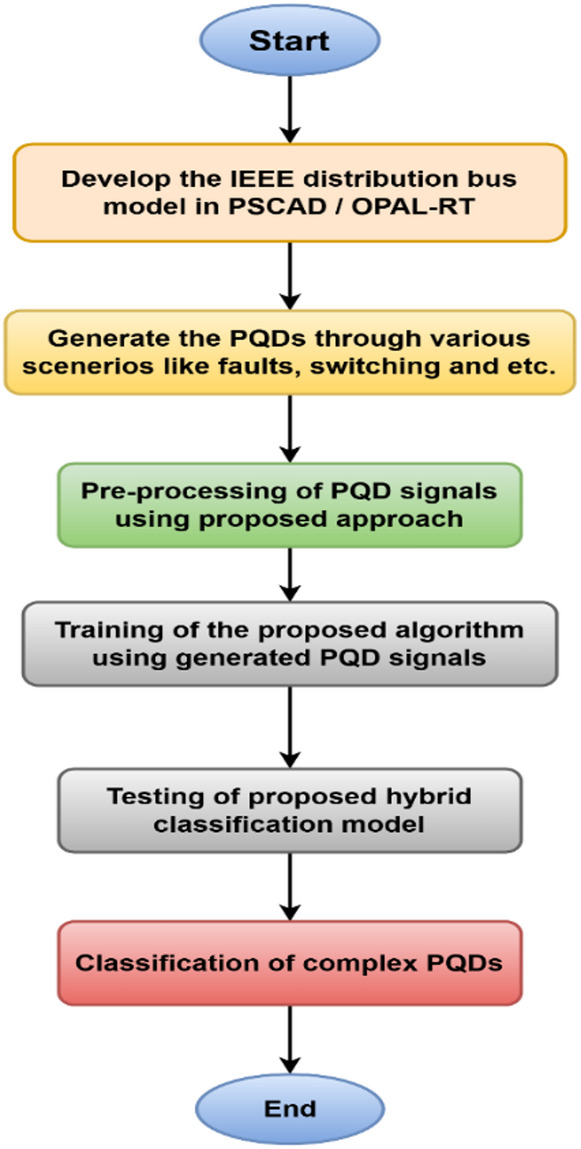
Work Flow of the suggested approach.

The LSTM cell receives the input (X_t_) and the output (h_t−1_) from the previous time step. Prior to entering the gates, the input (X_t_) and the previous hidden state (h_t−1_) are integrated in sequence. The node’s next input, originating from the previous node, is depicted as a straight line traversing the upper most layer of the node. The node state enables the LSTM to retain long-term dependencies while significantly mitigating the risks of vanishing and ballooning gradient issues that commonly afflict standard RNNs^23^. Figure 2 illustrates the Work flow of the suggested approach.

## Results and discussions

This part contains the discussions on the results and the entire simulations and implementation of suggested methodology real-time environment. The software execution is performed on an windows 10 PC provided with Intel Core i7 processor (3.6 GHz), 16 GB DDR4 RAM and an NVIDIA GeForce GTX1660 Super GPU. The Software packages include MATLAB/Simulink 2022a (https://in.mathworks.com/products/new_products/release2022a.html), PSCAD 4.5 (https://www.pscad.com/software/pscad/version-comparison). The real-time environment includes an OPAL-RT 4510 simulator mated with RT-LAB 2019 software (https://www.opal-rt.com/software-platforms/) for real-time validation. The detailed information about the software/tools used in this study are referred in the Sects^37–40^.

To validate the efficacy of the suggested hybrid classifier methodology, the subsequent situations of disturbance signals have been examined:

(i) Six different types of single disturbances.
(ii) Seven different types of simultaneously occurring double disturbances.
(iii) Three different types of simultaneously occurring triple disturbances.
(iv) Two different types of simultaneously occurring quadruple disturbances.

Further, the above-mentioned scenarios have been simulated considering the following systems: (i) PQD signals analytically generated using mathematical modelling as per IEEE standard (ii) PQDs created on simulating real world disturbance scenarios on IEEE 9 and 33 bus systems in PSCAD, (iii) to validate the proposed hybrid approach in real time environment, PQDs on IEEE 9 and 33 bus systems has also been simulated in OPAL-RT.

Here, 18,000 signals are created, comprising 1000 signals for each class of the 18 PQDs including 1000 normal signals, in accordance with IEEE standard 1159. The efficacy of the suggested method in addressing noise is demonstrated using contaminating signals with white gaussian noise of signal-to-noise ratio between 20 dB and 40 dB. 70% of the data is allocated for training each class, while the remaining 30% is designated for testing. The outcomes for each scenario and system are detailed in the following sections.

(i) **PQDs testes on synthetic signals**.

In this instance, several PQ disturbance signals are produced utilizing the mathematical formulas presented in Table 1, with a sample frequency of 6.4 kHz and an operating frequency of 50 Hz.

**Table 1:** Mathematical modeling of various PQDs.

PQDs	Mathematical Equations	Parameters
Normal	$\:x\left(t\right)={V}_{max}*\mathrm{S}\mathrm{i}\mathrm{n}\left(2\pi\:ft\right)$	$\:{V}_{max}=1.0$; $\:f=50\:Hz$
Sag	$\:x\left(t\right)={V}_{max}*\mathrm{S}\mathrm{i}\mathrm{n}\left(2\pi\:ft\right)$	$\:{0.1\le\:V}_{max}\le\:0.9;\:\:f=50\:Hz$
Swell	$\:x\left(t\right)={V}_{max}*\mathrm{S}\mathrm{i}\mathrm{n}\left(2\pi\:ft\right)$	$\:{1.1\le\:V}_{max}\le\:1.8;\:\:f=50\:Hz$
Interruption	$\:x\left(t\right)={V}_{max}*\mathrm{S}\mathrm{i}\mathrm{n}\left(2\pi\:ft\right)$	$\:{0\le\:V}_{max}\le\:0.1;\:\:f=50\:Hz$
Transient	$$\:x\left(t\right)={V}_{max}*\mathrm{Sin}\left(2\pi\:ft\right)+{V}_{max}*k*{e}^{-\frac{t}{\tau\:}}*\mathrm{S}\mathrm{i}\mathrm{n}\left(2\pi\:{f}_{n}t\right)$$	$$\:{V}_{max}=1.0$$; $$\:f=50\:Hz$$; $$\:0.1\le\:k\le\:0.8;$$ $$\:150\le\:\tau\:\le\:1000;$$ $$\:700\:Hz\le\:{f}_{n}\le\:1600\:Hz$$
Flicker	$\:x\left(t\right)={V}_{\mathrm{m}\mathrm{a}\mathrm{x}}*[1+\mathrm{Sin}\left(2\pi\:f\delta\:t\right)]*\mathrm{S}\mathrm{i}\mathrm{n}\left(2\pi\:ft\right)$	$\:{V}_{max}=1.0$; $\:f=50\:Hz$; $\:0.1\le\:\delta\:\le\:0.4$
Harmonics	$\:x\left(t\right)={V}_{max}*\mathrm{Sin}\left(2\pi\:ft\right)+\sum\:_{n=1}^{N}{V}_{n,max}*\mathrm{S}\mathrm{i}\mathrm{n}\left(2\pi\:\right(2n+1\left)ft\right)$	$\:{V}_{max}=1.0$; $\:f=50\:Hz$; $\:0.01\le\:{V}_{n,max}\le\:0.2$

**Fig. 3:**
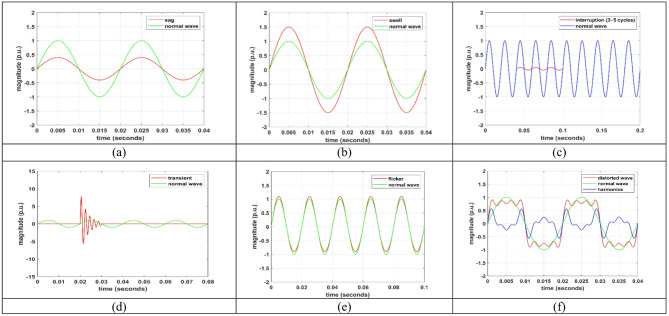
Various types of PQ disturbances. (**a**) Sag. (**b**) Swell. (**c**) Interruption. (**d**) Impulsive Transient. (**e**) Flicker. (**f**) Harmonics.

The simultaneous appearance of the above-mentioned disturbances, specifically the amalgamation of any two or more, can be represented by aggregating the respective mathematical equations. Consequently, elevated tiers of PQDs can be attained to enhance the system’s adaptability. Figures 3 (a)-(f) illustrate the graphs acquired for different PQDs.

S-Transform has been applied on the generated signals to extract the various features such as Root Mean Square (RMS), peak magnitude, S-Transform based power, S-Transform based phase angle, shape factor, crest factor, normal deviation. Chi-square test has been performed to select suitable features. After that those features are used carry out the training of the LSTM algorithm. The classes for the LSTM algorithm related to the specified PQ disturbances are presented in Table 2.

**Table 2:** Classes of PQDs as input.

Power Quality Disturbances	Classes	Power Quality Disturbances	Classes
Sag	PQ1	Transient + Harmonics	PQ10
Swell	PQ2	Flicker + Harmonics	PQ11
Interruptions	PQ3	Sag + Transient	PQ12
Impulsive Transient	PQ4	Swell + Transient	PQ13
Flicker	PQ5	Sag + Harmonics + Transient	PQ14
Harmonics	PQ6	Swell + Harmonics + Transient	PQ15
Sag + Harmonics	PQ7	Flicker + Transient + Harmonics	PQ16
Swell + Harmonics	PQ8	Sag + Transient + Flicker + Harmonics	PQ17
Interruptions + Harmonics	PQ9	Swell + Transient + Flicker + Harmonics	PQ18

**Fig. 4:**
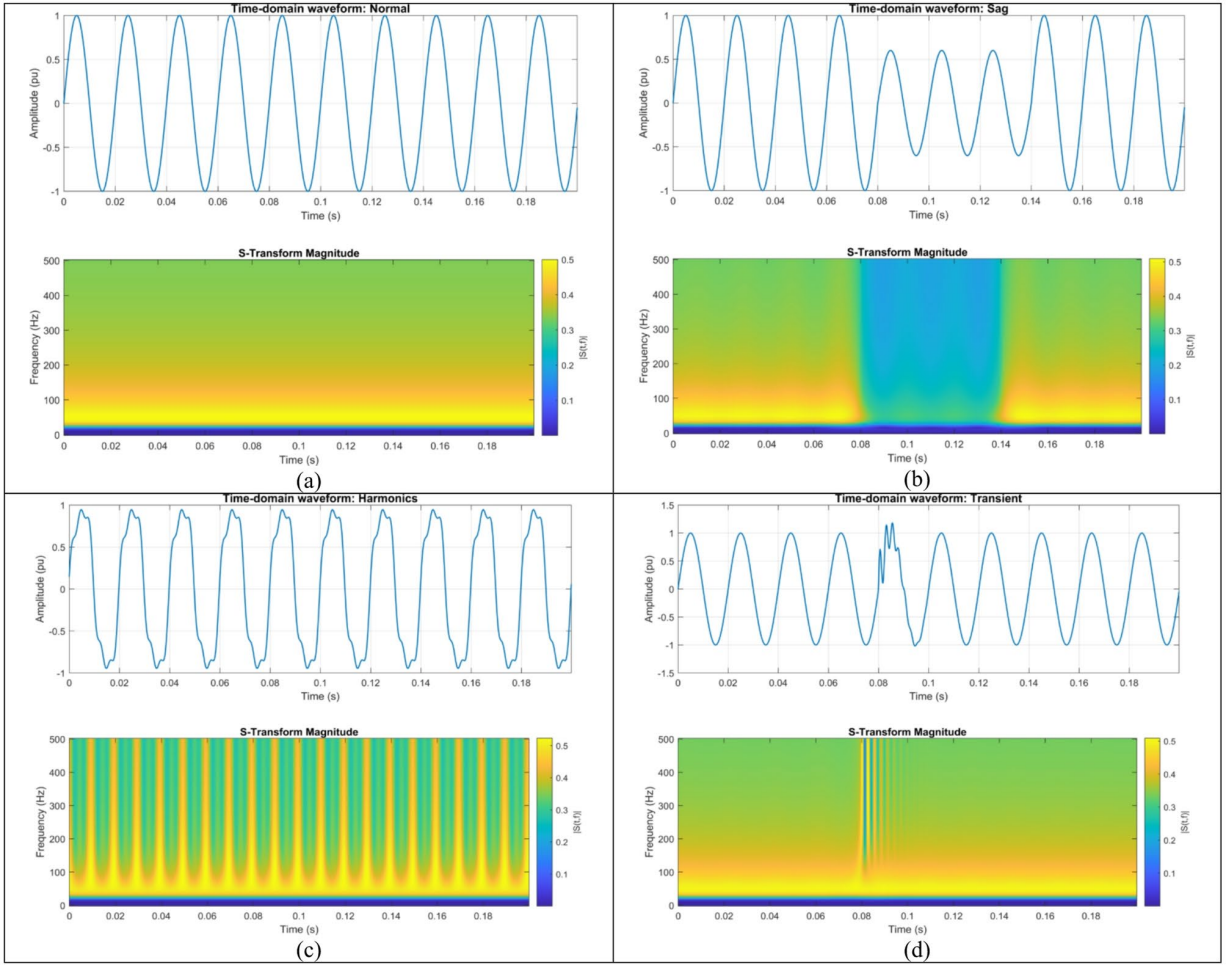
Visualizations of the time domain PQD signals along their time-frequency mapping image.

To illustrate how various disturbances exhibit distinct spectral patterns that are accurately captured by the S-Transform, input PQD signals in time domain alongside their respective S-Transform time-frequency maps are given in Fig. 4 (a-d). In the figure, we have shown only 04 classes as a sample for better understanding. The Fig. 4(a) represents the normal signal, which has found no significant patterns or disturbances in the s-transform based colour coded time-frequency map. The sag signal, Fig. 4(b), exhibit distinct amplitude diminutions and augmentations in the both time domain and energy distribution of the S-Transform. The transient signal, Fig. 4(d), displays localized high-frequency bursts at approximately 0.08 s, whereas harmonic-rich signals, Fig. 4(c), reveal several frequency ridges at 150 Hz, 250 Hz, and so on. The numerical values of statistical features ( as a sample for understanding of the researchers) such as RMS, peak magnitude, S-Transform power, mean phase angle, shape factor, crest factor, and normal deviation retrieved from each S-Transform output are shown in Table 3. The Chi-Square Test was employed to analyze the statistical coefficients and to select the significant features as inputs to the LSTM. The results unequivocally indicate that the proposed S-Transform-based feature extraction, Chi-Square Test based feature selection and LSTM sequence learning, facilitates precise and resilient identification of both single and hybrid PQDs.

**Table 3:** Features extractions variables from the PQD signals.

Class Label	RMS	Peak Magnitude	S-Transformed based Power	Mean Phase	Shape Factor	Crest Factor	Normal Deviation
Sag	0.4118	0.8057	0.1695	-3.0086	1.1140	1.9569	0.1815
Swell	0.5032	1.0474	0.2532	-0.7111	1.0995	2.0814	0.2092
Harmonics	0.4659	0.7617	0.2171	0.0166	1.0638	1.6346	0.1589
Transient	0.4592	0.9557	0.2109	-0.0911	1.0912	2.0811	0.1844
Flicker	0.4683	0.9642	0.2193	-0.0415	1.1017	2.0591	0.1965
Sag + Harmonics	0.4188	0.7620	0.1754	-0.6194	1.0862	1.8193	0.1635

To propose the efficient hybrid classifier for classification of complex PQDs, the performance of the hybrid approach comprise of ST-LSTM is contrasted with the hybrid approach comprised of ST-MLFFNN on the basis of computational time, prediction error, regression and gradient. The findings are presented in Table 4.

**Table 4:** Performance comparison between ST-MLFFNN and ST-LSTM.

	ST-MLFFNN	ST-LSTM
Iteration	5000	5000
Computation Time	00:27:17	00:34:10
Mean Square Error	2.77*10^− 6^	5.50*10^− 8^
Regression curve	99.24%	99.67%
Gradient	1.07*10^− 6^	2.99*10^− 6^

From the results, it can be derived that in comparison to MLFFNN model, the LSTM model has more complexity and hence, more computation time. On the other hand, the regression i.e. the fitting of the sample points gets improved and mean square error decreases in LSTM. From the performance comparison of the discussed approaches, it can be concluded that choose MLFFNN model where precision doesn’t matter, however, for better fitting and higher accuracy LSTM is better.

The comparative performance of the proposed methodology alongside MLFFNN across all four scenarios, both with and without noise, is presented in Table 5. The results indicate that the proposed technique outperforms the MLFFNN-based approach across all scenarios, both in noise-free environments and in conditions with noise levels of 20 dB and 40 dB. For scenario-i to iv, the average accuracy of proposed algorithm is lying between 99.5 and 96.4 for noise free environment while in noisy environment it is varying between 97.8 and 96.2 for 40 dB and 97.2 to 95.6 for 20 dB.

**Table 5:** Performance comparison of ST-MLFFNN and ST-LSTM on synthetic signals.

Scenarios	Noise	Avg. Accuracy (%) using ST-MLFFNN	Avg. Accuracy (%) using ST-LSTM
Single PQDs	Noise free	97.5	99.5
	40 dB	96.3	97.8
	20dB	95.7	97.2
Double PQDs	Noise free	95.4	98.4
	40 dB	95.0	97.4
	20dB	94.9	96.9
Triple PQDs	Noise free	89.3	96.6
	40 dB	89.1	96.2
	20dB	88.7	95.5
Quadruple PQDs	Noise free	88.0	96.4
	40 dB	87.9	96.2
	20dB	86.9	95.6

**Fig. 5:**
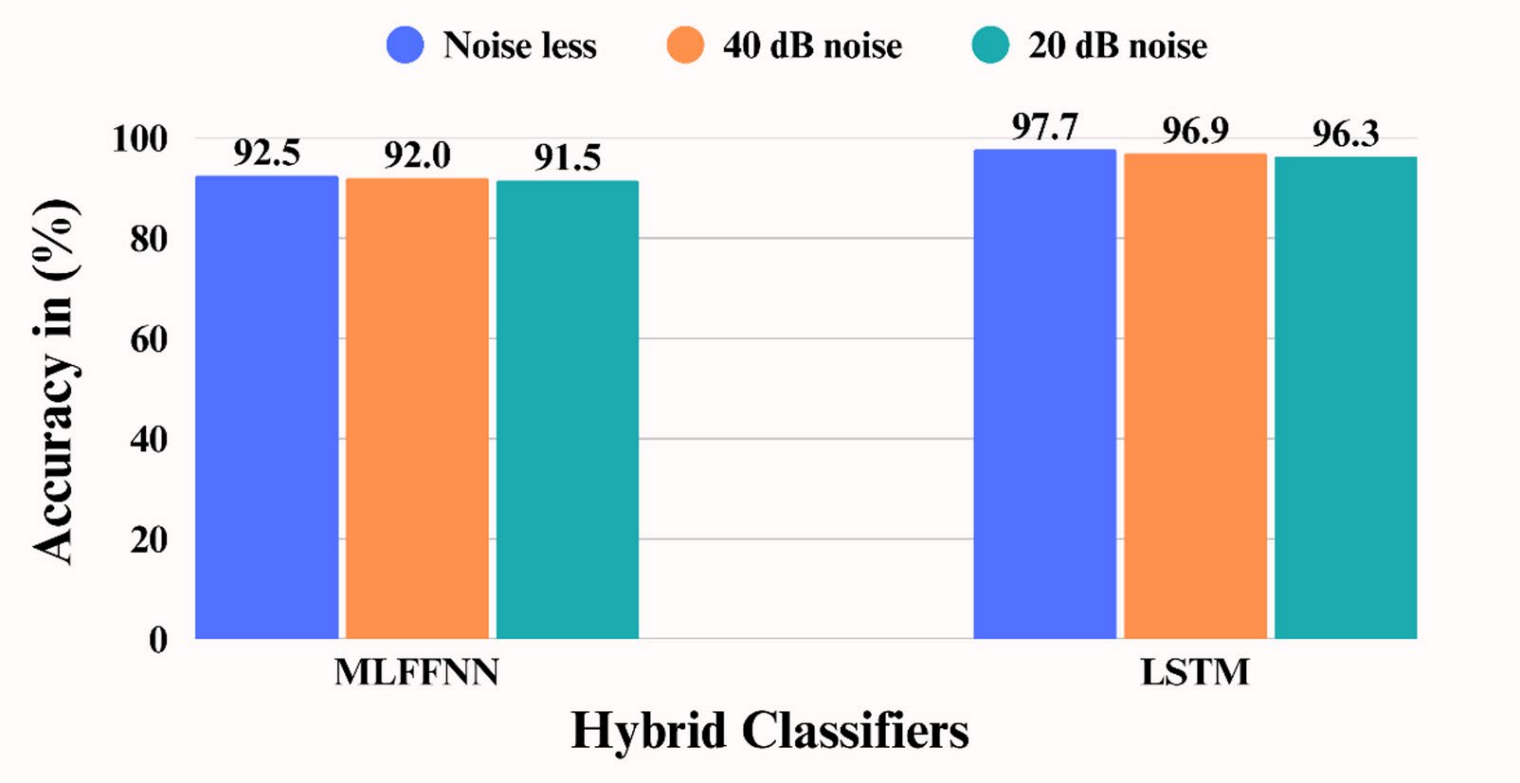
Performance accuracy for various noise levels.

The performance comparison of LSTM and MLFFNN considering average accuracy for all the scenarios under noise free as well as noisy environment (20 & 40 dB) is illustrated in Fig. 5. From the evaluation of the results, this is found that the suggested hybrid approach is capable to classifying the complex PQDs more precisely.

(ii) **PQDs created on IEEE-9 and IEEE-33 bus systems in PSCAD**

In order to validate the feasibility of the suggested ST-LSTM based hybrid approach, IEEE 9 and 33 bus distribution systems have been modelled in PSCAD simulation platform. The simulation environment consists of multiple (Distributed generators) DGs and many categories of load comprise of resistive-inductive-capacitive and nonlinear loads, power electronics appliances and shunt capacitor bank. Single and simultaneously occurring double PQDs have been recorded through faults and switching activities and simulating test system in special operating scenarios. Details of the disturbances are given in Table 2. Methodology discussed in section-2 has been implemented to classify the disturbances. The classification results obtained using proposed methodology is given in the Table 6.

**Table 6:** Performance comparison of ST-MLFFNN and ST-LSTM on disturbances created in PSCAD.

PQDs	Accuracy (%) using ST-MLFFNN		Accuracy (%) using ST-LSTM	
	IEEE 9- bus	IEEE 33- bus	IEEE 9- bus	IEEE 33- bus
PQ1	99.00	99.00	100.00	100.00
PQ2	98.00	96.00	100.00	100.00
PQ3	98.00	98.00	100.00	100.00
PQ4	99.00	95.00	99.00	99.00
PQ5	95.00	93.00	97.00	95.00
PQ6	97.00	95.00	99.00	99.00
PQ7	97.00	93.00	99.00	97.00
PQ8	95.00	94.00	99.00	99.00
PQ9	93.00	90.00	99.00	97.00
Average accuracy (%)	**96.78**	**94.77**	**99.11**	**98.44**

From the classification results, it is seen that the average accuracy of proposed hybrid approach is better compared to MLFFNN based hybrid approach for both the IEEE systems under noisy as well as noise free environment.

(iii) **PQDs on IEEE-9 and IEEE 33 bus systems simulated in OPAL-RT**

**Fig. 6:**
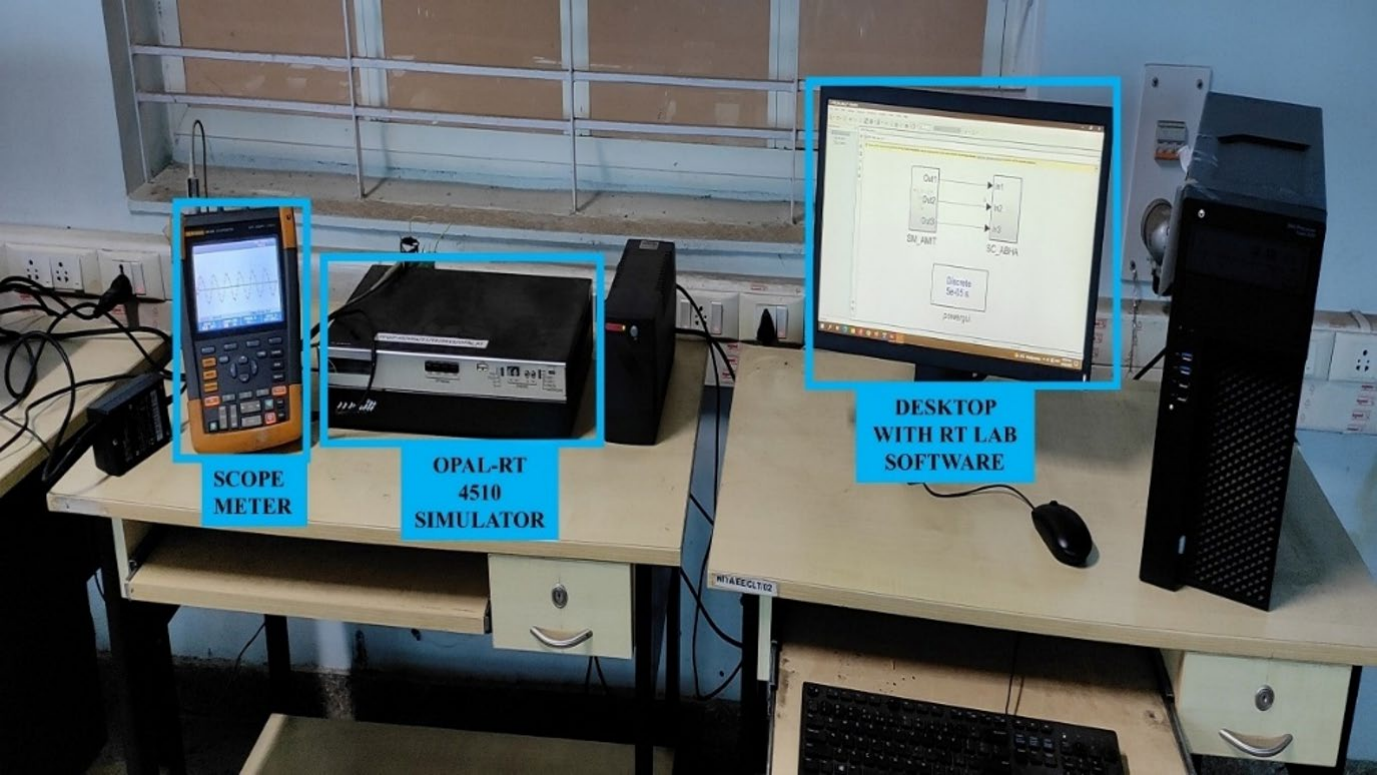
Experimental set up for real time validation.

To show the practical implementation of the proposed ST-LSTM based hybrid classifier, finally, the suggested method has been validated and tested with near to real time disturbance signals using IEEE-9 and 33 bus models in OPAL-RT system. Multiple disturbance signals are generated in the OPAL-RT system and discretized & processed using S-Transform. The experimental set up for the validation has been visualized in Fig. 6. The classification accuracy of various PQDs is listed in Table 7.

**Table 7:** Performance comparison of ST-MLFFNN and ST-LSTM on PQDs in OPAL-RT.

PQDs	Accuracy (%) using ST-MLFFNN		Accuracy (%) using ST-LSTM	
	IEEE 9- bus	IEEE 33- bus	IEEE 9- bus	IEEE 33- bus
PQ1	99.00	97.00	100.00	100.00
PQ2	98.00	94.00	99.00	99.00
PQ3	98.00	96.00	99.00	97.00
PQ4	96.00	94.00	100.00	98.00
PQ5	94.00	92.00	99.00	95.00
PQ6	97.00	93.00	98.00	98.00
PQ7	96.00	94.00	97.00	95.00
PQ8	96.00	92.00	99.00	97.00
PQ9	95.00	91.00	98.00	96.00
Avg. accuracy (%)	**96.56**	**93.67**	**98.78**	**97.22**

From the results given in Table 6, it is observed that the classification performance of ST-LSTM is higher compared to ST-MLFFNN in noisy as well as in noise free environment. However, in this case, the average accuracy has been reduced compared to scenario-ii. For ready reference, the comparison of the obtained accuracies in scenarios- i & ii has been shown in the Table 8.

**Table 8:** Performance comparison between ST-MLFFNN and ST-LSTM in PSCAD and OPAL-RT.

System	ST-MLFFNN Performance (%)		ST-LSTM Performance (%)	
	IEEE 9-bus	IEEE 33-bus	IEEE 9-bus	IEEE 33-bus
PSCAD	96.78	94.77	99.11	98.44
OPAL-RT	96.56	93.67	98.78	97.22

To further demonstrate the performance of the ST-LSTM approach, the results obtained using proposed hybrid approach (ST-LSTM) have been compared with the results reported for some of the recent approaches. The same are displayed in Table 9.

**Table 9:** Performance comparison of proposed hybrid approach with the recent approaches.

Methodology, [Refs. article], (Year)	Environment	Average accuracy (%)
ST and PNN^25^, (2008)	Noise free	97.2
	Noisy	93.2
KF & Fuzzy^26^, (2012)	Noise free	98.71
	Noisy	97.00
ST & dynamic HM^24^, (2013)	Noise free	99.2
	Noisy	94.2
Adaline and FFNN^27^, (2014)	Noise free	97.54
	Noisy	90.63
SSD and DT^28^, (2015)	Noise free	97.07
	Noisy	97.2
TQWT with Dual MSVM-RBF^29^, (2018)	Noise free	98.78
	Noisy	96.42
DWT-RF^41^, (2020)	Noise free	96.48
	Noisy	-----
SSPQDD^43^, (2022)	Noise free	96.55
	Noisy	-----
CNN-based DL^30^, (2023)	Noise free	94.71
	Noisy	94.54
URPM-CWT^42^, (2024)	Noise free	97.7
	Noisy	-----
**Proposed Hybrid Approach**	**Noise free**	**98.61**
	**Noisy**	**96.75**

It can be observed that the accuracy of all methods is higher for noise free signals whereas in noisy environment it is less. Based on the evaluation, it is observed that the suggested approach is more capable to recognize highly complex PQDs (simultaneously occurring up to quadruple disturbances), compared to the other approaches (considered simultaneously occurring up to double PQDs only). In contrast to alternative methods, the decline in accuracy of the suggested methodology is minimal for the majority of disturbances, despite its considerable complexity, which is a significant advantage.

## Conclusion

This paper proposes a hybrid amalgamation of S-Transform-CST-LSTM algorithm for the classification of quadruple PQDs occurring simultaneously. In the first stage, the proposed algorithm implements the S-Transform for feature extraction from the input PQD signals. From these feature vectors, the significant features are chosen by chi-square test. The S-Transform and Chi-Square methods have been employed to determine the optimal number of features that represent the PQDs, thereby assisting the classifier in improving its classification performance. In the second stage, LSTM algorithm is used for automatic classification of complex PQD signals up to quadruple. The results obtained LSTM classifier shows that the proposed algorithm is well suited for automatic classification of highly complex simultaneously occurring PQDs. The outcomes achieved with the proposed classifier have been compared with those from the MLFFNN-based classifier and other existing AI approached classifiers outlined in the manuscript. The authentication of experimented disturbance signals produced from IEEE distribution systems modelled in PSCAD and OPAL-RT which validates the effectiveness of the proposed ST-LSTM hybrid classifier in detecting and recognizing power quality disturbances in a real-time environment.

The proposed technique is limited by its necessity for a substantial dataset to achieve effective feature learning during training. As the world transitions to distributed energy supplies, new and more complex PQD signals may arise. Consequently, considering the intricacies of complex PQDs, a methodology has been developed to manage the concurrent complicated PQD signals. In future, the suggested algorithm will be implemented in real time using cost-effective embedded microcontrollers such as Raspberry Pi, and DSP boards with IoT functionalities. Moreover, explainable AI will be employed to obtain insights and assess the reliability of models.

## Data Availability

Data sets generated during the current study are available from the corresponding author on reasonable request.
